# Dapagliflozin alleviates renal inflammation and protects against diabetic kidney diseases, both dependent and independent of blood glucose levels

**DOI:** 10.3389/fimmu.2023.1205834

**Published:** 2023-11-09

**Authors:** Anxiang Cai, Jianxiao Shen, Xiaoqian Yang, Xinghua Shao, Leyi Gu, Shan Mou, Xiajing Che

**Affiliations:** Department of Nephrology, Molecular Cell Lab for Kidney Disease, Ren Ji Hospital, Shanghai Jiao Tong University School of Medicine, Shanghai, China

**Keywords:** diabetic kidney disease, sodium-glucose cotransporter 2 inhibitors, inflammation, renal tubular cell, macrophage

## Abstract

**Introduction:**

Diabetic kidney disease (DKD) has become the leading cause of end-stage renal disease worldwide. Therefore, efforts to understand DKD pathophysiology and prevent its development at the early phase are highly warranted.

**Methods:**

Here, we analyzed kidneys from healthy mice, diabetic mice, and diabetic mice treated with the sodium-glucose cotransporter 2 inhibitor dapagliflozin using ATAC and RNA sequencing. The findings were verified at the protein levels and in cultured cells.

**Results:**

Our combined method of ATAC and RNA sequencing revealed *Csf2rb*, *Btla*, and *Isg15* as the key candidate genes associated with hyperglycemia, azotemia, and albuminuria. Their protein levels were altered together with multiple other inflammatory cytokines in the diabetic kidney, which was alleviated by dapagliflozin treatment. Cell culture of immortalized renal tubular cells and macrophages unraveled that dapagliflozin could directly effect on these cells *in vitro* as an anti-inflammatory agent independent of glucose concentrations. We further proved that dapagliflozin attenuated ischemia/reperfusion-induced chronic kidney injury and renal inflammation in mice.

**Discussion:**

Overall, our data emphasize the importance of inflammatory factors to the pathogenesis of DKD, and provide valuable mechanistic insights into the renoprotective role of dapagliflozin.

## Introduction

With the increasing prevalence of diabetes mellitus, diabetic kidney disease (DKD) has become the leading cause of end-stage renal disease (ESRD) worldwide, burdening approximately half of the patients with type 2 diabetes mellitus and one-third with type 1 diabetes mellitus (1, 2). Patients with DKD naturally undergo glomerular hyperfiltration, progressive albuminuria, and glomerular filtration rate decline before finally developing ESRD (2). Undoubtedly, efforts to explore DKD pathophysiology and prevent its progression at the early phase would benefit DKD patients, improving their life quality and prognosis.

Previous studies to investigate the pathomechanism of DKD unraveled the effects of metabolic and hemodynamic factors on intrinsic renal cells, including glomerular endothelial cells, mesangial cells, podocytes, and tubular cells (2, 3). However, emerging evidence has proved that systemic and local renal inflammation plays a crucial role in the pathogenesis of DKD (4). Among various inflammatory cells, macrophage infiltration is apparent in the DKD glomerulus, and its activation is closely associated with the severity of the disease (5).

Inhibition of the sodium-glucose cotransporter 2 (SGLT2) in the renal proximal tubular cells represents an effective anti-hyperglycemic strategy, which also benefits the kidney and cardiovascular function of diabetic patients (6, 7). Dapagliflozin is a promising SGLT2 inhibitor with broad clinical application, which can benefit patients through glucose lowering, weight loss, and blood pressure reduction. In the long term, dapagliflozin has been demonstrated to sustain the estimated glomerular filtration rate, and reduce the risk of ESRD and kidney-related or cardiovascular death in patients with DKD or non-diabetic chronic kidney diseases (CKD) (8–10). Current mechanistic understanding of the renoprotective effect of SGLT2 inhibitors focused on renal microvascular hemodynamics and renal intrinsic cell metabolism (6, 7), whereas how this therapy could regulate renal inflammation remains largely unclear.

In this study, we performed RNA sequencing to screen for candidate genes of DKD that respond to dapagliflozin treatment *in vivo*. In combination with cell culture *in vitro*, we demonstrated that inflammatory molecules, such as colony-stimulating factor 2 receptor subunit beta (CSF2RB), B and T lymphocyte attenuator (BTLA), and interferon-stimulated gene 15 (ISG15), should play pivotal roles in DKD. In addition to its anti-hyperglycemic function, dapagliflozin could directly affect renal proximal tubular cells and macrophages to exert anti-inflammatory function and protect against DKD.

## Materials and methods

### Animals and tissue collection

The animal experiments were performed in the Experimental Animal Platform of Renji Hospital, and all animal research was approved by the Institutional Animal Ethics Committee of Renji Hospital. Briefly, 8-week-old male C57BL/6 mice and BKS.Cg-Dock7m +/+ Leprdb/J(db/db) mice were both purchased from SLAC Laboratory Animal Co., Ltd. (Shanghai, China). Mice were housed under controlled ambient conditions with standard chow, a 12-h light/dark cycle (lights on at 7 a.m.), and were maintained at 25°C. For experiments, mice were divided into three groups: control group (Ctrl, C57BL/6 mice, n=5), diabetic group (Case, db/db mice, n=6), and treatment group (Dapa, db/db mice treated with dapagliflozin [1.0 mg/kg/day, AstraZeneca, Cambridge, UK], n=6) for 6 weeks. Dapagliflozin was administrated in the drinking water by oral gavage once daily in the treatment group, and mice in the Ctrl and Case groups were administrated with the same volume of saline. When the experiment was completed, all mice in the three groups were sacrificed by cervical dislocation after CO_2_-induced narcosis. Immediately afterward, the kidneys and blood were collected.

For ischemia/reperfusion-induced chronic kidney disease mouse models, 14-week-old male C57BL/6 mice were anesthetized and subjected to right kidney nephrectomy. Right kidneys were collected and used as controls, and the left renal artery was clamped for 30 minutes at 37°C followed by reperfusion. At 7 days post surgery, kidney tissues and blood and urine samples were collected for further analysis.

### RNA sequencing

Total RNA was isolated using an RNeasy mini kit (Qiagen, Germany). Strand-specific libraries were prepared using the TruSeq Stranded Total RNA Sample Preparation kit (Illumina, USA), following the manufacturer’s instructions. Briefly, mRNA was enriched with oligo(dT) beads. Following purification, the mRNA is fragmented into small pieces using divalent cations under 94°C for 8 min. The cleaved RNA fragments are copied into first-strand cDNA using reverse transcriptase and random primers. This is followed by second-strand cDNA synthesis using DNA Polymerase I and RNase H. These cDNA fragments then go through an end repair process, adding a single’A’base, and then ligating the adapters. The products are then purified and enriched with PCR to create the final cDNA library. Purified libraries were quantified by Qubit 2.0 Fluorometer (Life Technologies, USA) and validated by Agilent 2100 bioanalyzer (Agilent Technologies, USA) to confirm the insert size and calculate the mole concentration. The clusters were generated by cBot with the library diluted to 10 pM and then were sequenced on the Illumina NovaSeq 6000 (Illumina, USA). The library construction and sequencing were performed at Shanghai Biotechnology Corporation.

### Analysis of RNA sequencing data

For each sample, 33-95M RNA-sequencing (RNA-seq) clean reads were obtained that mapped to the mouse reference genome (mm10) using hierarchical indexing for spliced alignment of transcripts (HISAT2) v2.0.477. Sequencing read counts were calculated using Stringtie78,79 (v.1.3.0). Then, expression levels from different samples were normalized by the Trimmed Mean of M values (TMM) method. The normalized expression levels of different samples were converted to Fragments Per Kilobase of transcript per Million mapped fragments (FPKM). The edgeR package of R was used to analyze the difference between intergroup gene expression, the P-values were calculated, and multiple hypothesis tests were performed. The P-value threshold was determined by controlling the False Discovery Rate (FDR) with the Benjamini algorithm. The corrected P-value is called the q-value. Differentially expressed genes (DEGs) were defined as transcripts with a fold change in expression level (according to the FPKM value) greater than 2.0 and a q-value less than 0.05. Gene ontology (GO) and Kyoto Encyclopedia of Genes and Genomes (KEGG) enrichment analysis were performed with the clusterProfiler package of R, and the enrichment criteria included a q-value < 0.05. Heatmaps of specific genes were generated using the heatmap package of R. K-means analysis was performed with Euclidean distances in Multiple Experiment Viewer 4.9, and yielded 10 clusters based on input FPKM values (11).

### Library construction for ATAC-seq and sequencing procedures

Briefly, a total of 20,000 cells per sample was washed twice with 50 µL of cold Dulbecco’s Phosphate-Buffered Saline and re-suspended in 50 µL of lysis buffer [10 mM tris-HCl (pH 7.4), 10 mM NaCl, 3 mM MgCl2, and 0.1% (v/v) NP-40 substitute (Sigma-Aldrich, 11332473001)]. The suspension of nuclei was then centrifuged for 10 min at 500g at 4°C, followed by the addition of 50 µL of transposition reaction mix (10 µL of 5×TTBL buffer, 4 µL of TTE mix, and 36 µL of nuclease-free H2O) of TruePrep DNA Library Prep Kit V2 for Illumina (Vazyme Biotech, TD501). Samples were then incubated at 37°C for 30 min. DNA was isolated using the QIAquick PCR Purification Kit (QIAGEN, 28106). ATAC-seq libraries were first subjected to five cycles of pre-amplification using NEBNext High-Fidelity 2X PCR Master Mix (New England Biolabs, M0541S). To determine the suitable number of cycles required for the second round of PCR, the library was assessed by qPCR using NEBNext High-Fidelity 2X PCR Master Mix with SYBR Green I Nucleic Acid Gel Stain (Thermo Fisher Scientific, S7563) and then PCR amplified for the appropriate number of cycles. Libraries were purified with the QIAquick PCR Purification Kit. Library quality was checked using the High Sensitivity DNA Analysis Kit (Agilent, 5067-4626). Last, 2 X 150 paired-end sequencing was performed on an Illumina Nova seq (Illumina, San Diego, CA). The library construction and sequencing were performed at Shanghai Biotechnology Corporation.

### ATAC data processing and analysis

Alignment and peak-calling of ATAC sequencing data were performed using the ENCODE-DCC ATAC-Seq pipeline (https://github.com/ENCODE-DCC/atacseq-pipeline) (ENCODE Project Consortium, 2012). Briefly, data were aligned to the mouse reference genome (mm10) using Bowtie2. Aligned data were filtered for PCR duplicates, blacklisted regions, and shifted +4bp for the + strand and -5bp for the - strand. Peaks were called using MACS2. Differential peak calling was performed using Diffbind (DiffBind,http://bioconductor.org/packages/release/bioc/vignettes/DiffBind/inst/doc/DiffBind.pdf.). Motif enrichment was performed using Hypergeometric Optimization of the Motif EnRichment program (HOMER) version 4.1.

### Data analyses tools

Pathway enrichment analysis was performed on the DEGs using the Gene Ontology (www.geneontology.org) and the Kyoto Encyclopedia of Genes and Genomes (https://www.kegg.jp/) databases.

### Proteinuria, BUN, and Creatinine analysis

Mouse urine albumin concentration and urine creatinine concentration were measured using the Albumin Creatinine Ratio Assay Kit (ab241018) from Abcam. The excretion of albumin is expressed as the urinary albumin creatinine ratio (UACR). Urine albumin concentration was confirmed by loading 1 μl urine sample per lane on a sodium dodecyl sulfate-polyacrylamide gel for electrophoresis, followed by Coomassie blue staining. 1 and 5 μg bovine serum albumin (BSA) were loaded as controls for albuminuria. BUN and plasma creatinine levels were measured using a Liquid Urea Nitrogen Reagent Set and Creatinine Assay kit (Nanjing Jiancheng Bioengineering Institute, China) according to the manufacturer’s protocol.

### Histopathology analysis

Renal tissue harvested from animals was washed with 0.9% saline, fixed in 10% neutral buffered formalin, and then embedded in 10% paraffin. Sections (4 µm thick) were stained with periodic acid-Schiff staining (PAS) for further microscopic analysis. Three consecutive glomerular cross-sections from each sample were photographed by a nephropathologist, blinded to the source of the tissue, using a digital camera (Olympus DP74; Olympus America, Melville, New York), and imported into Image-Pro Plus (Media Cybernetics, Silver Spring, Maryland).

### Cell culture

Immortalized mouse macrophage cell line (RAW264.7) was cultured in Dulbecco’s modified Eagle’s medium (DMEM, HyClone) supplemented with 10% heat-inactivated fetal bovine serum (Gibco), 100 U/mL penicillin, and 100 μg/mL streptomycin (Sigma) at 37°C under 5% CO_2_. Immortalized human renal proximal tubular cell line (HK-2) was cultured in DMEM/F12 medium (HyClone) supplemented with 10% heat-inactivated fetal bovine serum (Gibco), 100 U/mL penicillin, and 100 μg/mL streptomycin (Sigma) at 37°C under 5% CO_2_.

Before experiments, cells were seeded onto 6-well plates to reach a confluence of 70% and then starved in serum-free DMEM overnight. Cells were then divided into three groups: for the osmotic control group (Ctrl), cells were cultured in DMEM medium containing 5mM glucose and 30mM mannitol; for the high-glucose group (Case), cells were cultured in DMEM medium containing 35mM glucose; for the dapagliflozin treatment group (Dapa), cells were cultured in DMEM medium containing 35mM glucose and 20µM dapagliflozin. Macrophages were incubated for 48 hours, and renal proximal tubular cells were incubated for 72 hours.

### Western blotting

Proteins from mice kidneys or cultured cells were extracted using RIPA lysis buffer supplemented with a cocktail of protease and phosphatase inhibitors. The total protein concentration was measured using a BCA assay, and equal amounts of protein were loaded on PAGE 4-20% gradient gels (GenScript) and transferred to PVDF membranes (Millipore). The primary antibodies were mouse anti-CSF2RB (Santa Cruz Biotechnology), rabbit anti-BTLA (Invitrogen), mouse anti-ISG15 (Santa Cruz Biotechnology), rabbit anti-SGLT2 (Proteintech), and mouse anti-β-actin (Cell Signaling Technology). Blots were developed using Omni ECL reagent (EpiZyme).

### RNA extraction and real-time PCR

Total RNA from cultured cells was extracted using TRIzol Reagent (Invitrogen). RNA quality was checked by controlling the OD at 260 and 280 nm. cDNA was synthesized from 1 µg RNA using the Hifair II 1st Strand cDNA Synthesis Super Mix (Yeasen, China). Real-time PCR was performed with the Roche Light Cycler 480 detection system using SYBR green PCR master Mix (Roche Diagnostics). Specific primers for target mRNAs (listed in the **Supplementary Materials**) were used for amplification, and a standard curve was generated for each targeted transcript. All relative expression levels were normalized to β-actin expression, and the results were analyzed using the ΔΔCt method.

### Antibody array assay and enzyme-linked immunosorbent assay

Mouse whole kidney lysate and cell culture medium supernatant of RAW264.7 were scanned using the QAM-INF-1 array (Raybiotech), and cell culture medium supernatant of HK-2 was scanned using the Quantibody Human TH17 array (Raybiotech) to obtain the raw data. Then, the Raybiotech software was used to remove the chip background and normalize the original data. Differentially expressed proteins (DEPs) between different groups were identified using the limma package (Version 3.42.2) in R software (RStudio, PBC, Boston, MA, USA). Individual p-values were calculated and converted to adjusted p-values (adj. p. val) for comparisons by false discovery rate correction of the Benjamini and Hochberg test. A cutoff point of adj. p. val < 0.05 and |fold change (FC)| > 1.2 were used to select DEPs. Then, the heatmap, and scatter plot of the DEPs were drawn using the ggplot2 package in R software. The expression levels of a specific protein of interest were determined by ELISA. The raw data were detailed in the **Supplementary Material**.

### Functional enrichment analyses and annotation of DEPs

The DEPs were uploaded to an online bioinformatics database, the Database for Annotation, Visualization, and Integrated Discovery (DAVID) version 6.8 Beta (https://david-d.ncifcrf.gov/), for enrichment analysis, including gene ontology (GO) pathways. The obtained results were visualized in the R ggplot2 package and a p-value of <0.05 was considered statistically significant.

### Statistical analysis

Data analysis was performed using GraphPad Prism software. Differences between two groups were analyzed using unpaired Student’s two-tailed t-test, and differences between more than two groups were analyzed using a one-way ANOVA test. Each experiment was replicated for more than three times. P values are presented as follows: *, P < 0.05; **, P < 0.01; ***, P < 0.001; NS, not significant.

## Results

### Dapagliflozin alleviated hyperglycemia and kidney injury in type 2 diabetic mice *in vivo*

**Figure 1:**
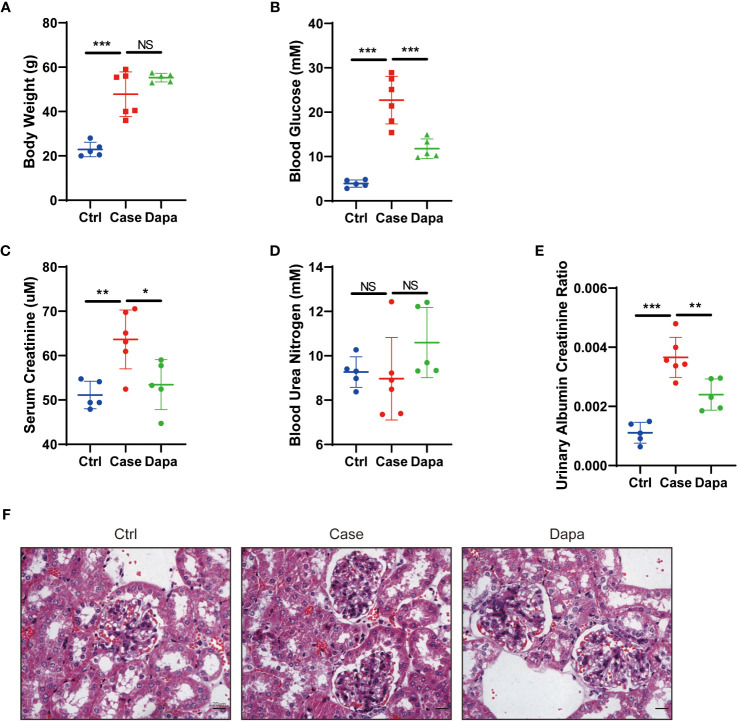
Dapagliflozin alleviated hyperglycemia and kidney injury in type 2 diabetic mice in vivo. **(A)** Body weight of normal (Ctrl), diabetic (Case), and dapagliflozin-treated diabetic (Dapa) 14-week-old mice. **(B)** Blood glucose levels of 14-week-old mice in the Ctrl, Case, and Dapa groups. **(C)** Serum creatinine levels of 14-week-old mice in the Ctrl, Case, and Dapa groups. **(D)** Blood urea nitrogen levels of 14-week-old mice in the Ctrl, Case, and Dapa groups. **(E)** Urinary albumin creatinine ratio of 14-week-old mice in the Ctrl, Case, and Dapa groups. **(F)** Periodic acid–Schiff (PAS)–stained sections of 14-week-old mouse kidneys in the Ctrl, Case, and Dapa groups. NS, not significant. *P < 0.05, **P < 0.01, ***P < 0.001, one-way ANOVA test. Bar = 20 μm.

Transgenic db/db mice (Case) of 14-week-old showed spontaneous type 2 diabetes mellitus phenotype: compared with the healthy mice (Ctrl), diabetic mice had augmented body weight (22.9 ± 2.9 g in Ctrl versus 47.8 ± 9.2 g in Case, P<0.0001) and hyperglycemia (3.9 ± 0.7 mM in Ctrl versus 22.7 ± 4.9 mM in Case, P<0.0001) (**Figures 1A, B**). In contrast, db/db mice given oral administration of 1 mg/kg dapagliflozin for 6 weeks (Dapa) presented significantly lower blood glucose levels (11.8 ± 2.0 mM in Dapa, P=0.0006 compared with Case), though their bodyweight displayed no significant changes (55.3 ± 1.7 g in Dapa, P=0.1854 compared with Case) (**Figures 1A, B**).

As for kidney functions, db/db mice presented higher serum creatinine levels (51.1 ± 2.8 µM in Ctrl versus 63.7 ± 6.0 µM in Case, P<0.0001) and elevated urinary albumin creatinine ratio (UACR, 0.0011 ± 0.0003 in Ctrl versus 0.0037 ± 0.0006 in Case, P<0.0001) (**Figures 1C–E**). Treatment with dapagliflozin remarkably ameliorated azotemia (serum creatinine=53.4 ± 5.0 µM in Dapa, P=0.0215 compared with Case) and albuminuria (UACR=0.0024 ± 0.0004 in Dapa, P=0.0059 compared with Case) in diabetic mice (**Figures 1C–E**). Periodic acid-Schiff staining of renal tissue from the Case group exhibited mild-to-moderate diabetic renal structural alterations, including mesangial area broadening, mesangial matrix increase, and mesangial hyperplasia, while these changes were alleviated in mice receiving dapagliflozin treatment (**Figure 1F**). In short, dapagliflozin successfully rescued both hyperglycemia and diabetic nephropathy in diabetic mice.

### Combined ATAC and RNA sequencing revealed potential candidate genes for diabetic kidney disease

**Figure 2:**
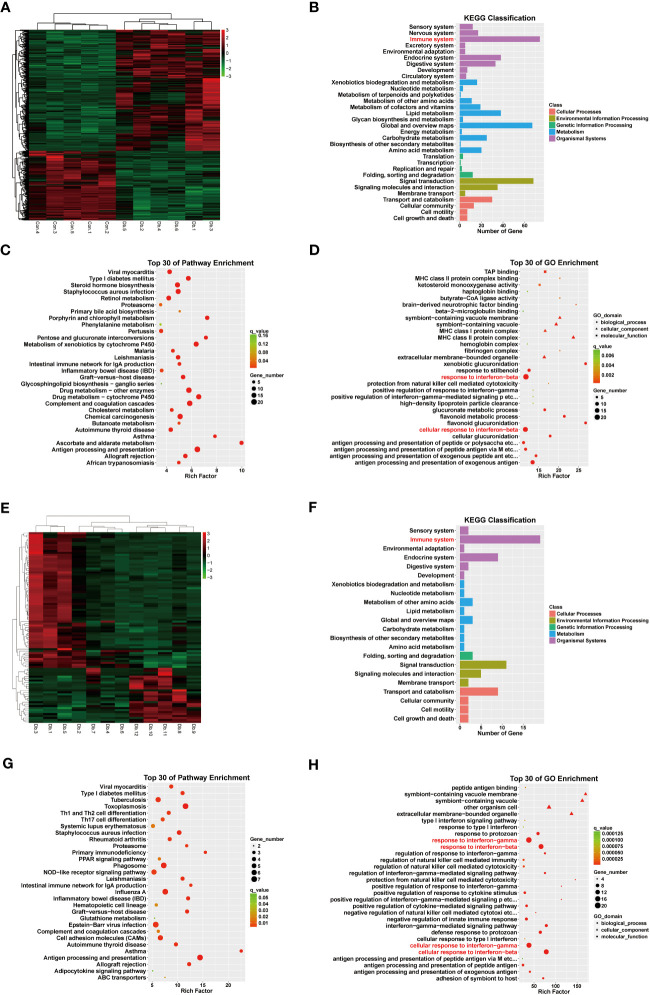
RNA sequencing revealed differentially expressed genes and pathways in diabetic kidney disease and dapagliflozin treatment. **(A)** Heatmap indicating distinct gene expression patterns of normal (Con1-5) versus diabetic (Db1-6) mouse kidneys. **(B)** KEGG classification analysis suggested that DEGs between normal and diabetic mouse kidneys were enriched in the immune system (red). **(C)** KEGG enrichment analysis indicating the top 30 pathways enriched in DEGs between normal and diabetic mouse kidneys. **(D)** GO enrichment analysis indicating the top 30 pathways enriched in DEGs between normal and diabetic mouse kidneys, highlighting interferon-related pathways (red). **(E)** Heatmap indicating distinct gene expression patterns of diabetic (Db1-6) versus dapagliflozin-treated diabetic (Db7-12) mouse kidneys. **(F)** KEGG classification analysis suggesting DEGs between diabetic and dapagliflozin-treated diabetic mouse kidneys were enriched in the immune system (red). **(G)** KEGG enrichment analysis indicating the top 30 pathways enriched in DEGs between diabetic and dapagliflozin-treated diabetic mouse kidneys. **(H)** GO enrichment analysis indicating the top 30 pathways enriched in DEGs between diabetic and dapagliflozin-treated diabetic mouse kidneys, highlighting interferon-related pathways (red).

To determine potential candidate genes that might participate in the pathogenesis of DKD, we performed a combined analysis of RNA sequencing and Assay for Transposase Accessible Chromatin with high-throughput sequencing (ATAC-seq) on mice whole kidney lysates. Mice displayed distinguished RNA expression patterns between the Ctrl group (Con.1-5) and the Case group (Db.1-6)(**Figure 2A**). Functional enrichment analysis utilizing the gene ontology (GO) database and Kyoto Encyclopedia of Gene and Genome (KEGG) database suggested that, in addition to metabolic disorders, diabetic mice showed immune system alterations that might involve response to interferon (**Figures 2B–D**). To be precise, analysis based on the KEGG database revealed pathways involving multiple material metabolism, inflammation and autoimmune-related diseases, and antigen processing and presentation (**Figure 2C**); while GO terms indicated material metabolism, antigen processing and presentation via MHC complex, and response to interferon (**Figure 2D**). Moreover, despite individual discrepancies, mice treated with dapagliflozin (Db.7-12) also presented different RNA expression patterns to the Case group (**Figure 2E**). Similarly, the top DEGs of these two groups were enriched in the immune-associated pathways, especially the response to interferon (**Figures 2F–H**). In the analysis of Dapa versus Case, the KEGG database unraveled pathways involving inflammation and autoimmune-related diseases, helper T cell differentiation, and antigen processing and presentation (**Figure 2G**); GO terms also suggested antigen processing and presentation, and response to interferon (**Figure 2H**).

**Figure 3:**
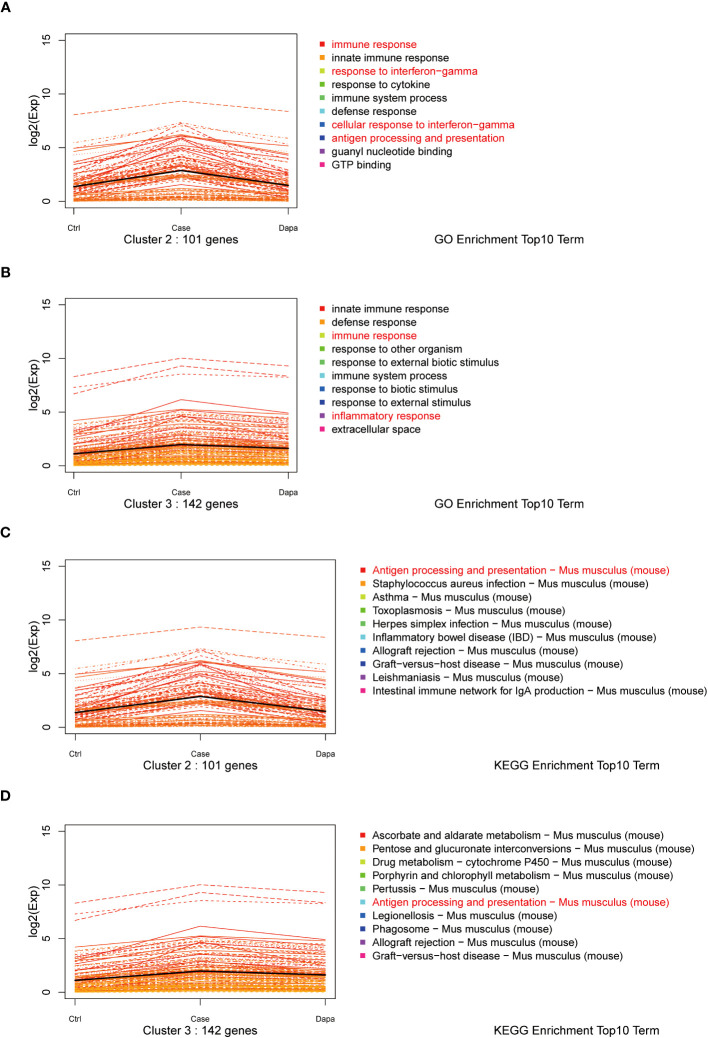
K-means analysis of RNA sequencing results. K-means analysis yielded 10 clusters with different expression patterns among the Ctrl, Case, and Dapa groups. Cluster 2 and 3 showed apparently different expression patterns among Ctrl, Case, and Dapa groups. **(A)** Expression patterns of genes in Cluster 2 with the top 10 enriched pathways listed according to GO terms. **(B)** Expression patterns of genes in Cluster 3 with the top 10 enriched pathways listed according to GO terms. **(C)** Expression patterns of genes in Cluster 2 with the top 10 enriched pathways listed according to the KEGG database. **(D)** Expression patterns of genes in Cluster 3 with the top 10 enriched pathways listed according to the KEGG database.

Furthermore, we performed a K-means analysis with the aim of revealing the co-ordinance between Ctrl and Dapa groups. According to their different expression patterns among the three groups, the DEGs were divided into 10 clusters. We noticed cluster 2 and cluster 3, in which DEGs showed upregulation in the Case group but downregulated by dapagliflozin treatment (**Figure 3**). GO terms and the KEGG database indicated that pathways enriched in these two clusters were immune response, response to interferon-gamma (IFN-γ), antigen processing and presentation, and inflammation-related diseases (**Figure 3**). In addition, DEGs in cluster 5 and cluster 9 also presented co-ordinance between Ctrl and Dapa groups: pathways enriched in cluster 5 mainly involved material metabolism, endocrine regulation, and renal intrinsic cells; while pathways enriched in cluster 9 were associated with immunoglobulin, PPAR, and FoxO pathways (**Supplemental Figure 1**).

**Figure 4:**
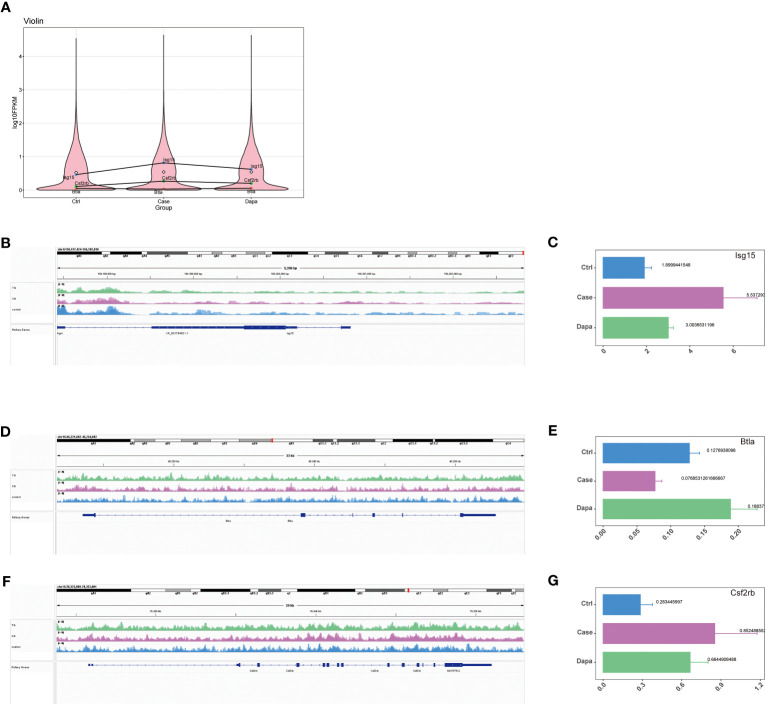
Combined ATAC and RNA sequencing revealed potential candidate genes for diabetic kidney disease. **(A)** Violin plot of RNA sequencing indicating *Isg15*, *Csf2rb*, and *Btla* as potential candidate genes that were upregulated in diabetic mouse kidneys and downregulated by dapagliflozin treatment (*Isg15* and *Csf2rb*), or downregulated in diabetic mouse kidneys and upregulated by dapagliflozin treatment (*Btla*). **(B)** ATAC analysis indicating elevated chromatin accessibility of *Isg15* in diabetic mouse kidneys that was reduced by dapagliflozin treatment, with corresponding quantifications in **(C)**. **(D)** ATAC analysis indicating decreased chromatin accessibility of *Btla* in diabetic mouse kidneys that was augmented by dapagliflozin treatment, with corresponding quantifications in **(E)**. **(F)** ATAC analysis indicating elevated chromatin accessibility of *Csf2rb* in diabetic mouse kidneys that was reduced by dapagliflozin treatment, with corresponding quantifications in **(G)**.

**Figure 5:**
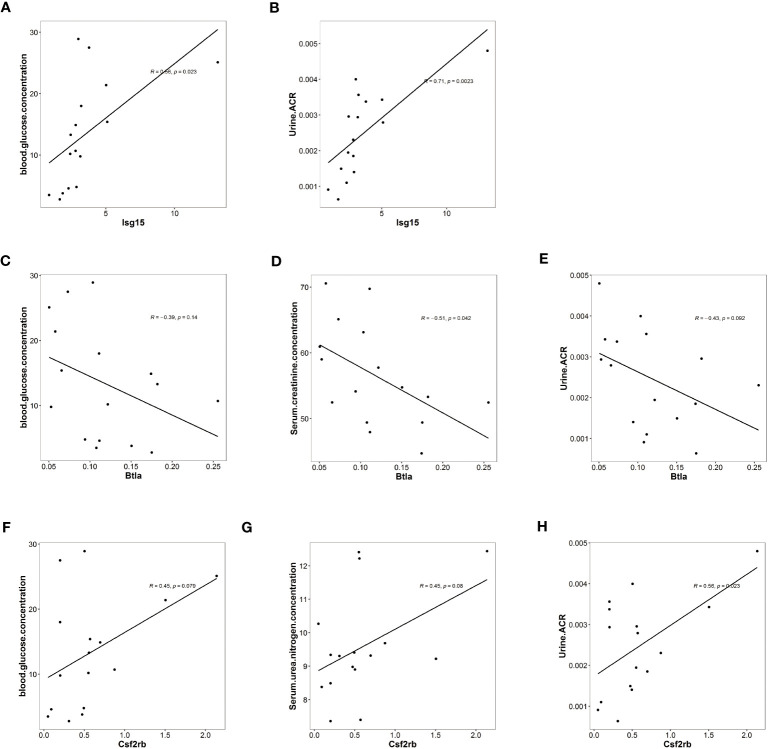
Isg15, Csf2rb, and Btla expression levels were associated with clinical features of diabetic kidney disease. **(A)** Univariate analysis demonstrating a positive correlation between *Isg15* expression levels and blood glucose levels. **(B)** Univariate analysis demonstrating a positive correlation between *Isg15* expression levels and urinary albumin creatinine ratio. **(C)** Univariate analysis demonstrating a negative correlation between *Btla* expression levels and blood glucose levels. **(D)** Univariate analysis demonstrating a negative correlation between *Btla* expression levels and serum creatinine levels. **(E)** Univariate analysis demonstrating a negative correlation between *Btla* expression levels and urinary albumin creatinine ratio. **(F)** Univariate analysis demonstrating a positive correlation between *Csf2rb* expression levels and blood glucose levels. **(G)** Univariate analysis demonstrating a positive correlation between *Csf2rb* expression levels and serum urea nitrogen levels. **(H)** Univariate analysis demonstrating a positive correlation between *Csf2rb* expression levels and urinary albumin creatinine ratio.

Combining the results of RNA sequencing and ATAC analysis, we identified three inflammation-associated genes that showed opposite regulation trends in Case versus Ctrl and Dapa versus Case, i.e., *Isg15*, *Btla*, and *Csf2rb* (**Figure 4**). *Isg15* (**Figures 4A–C**) and *Csf2rb* (**Figures 4A, F–G**) presented upregulated chromatin accessibility and mRNA levels in diabetic mice and attenuated by dapagliflozin administration. In contrast, *Btla* (**Figures 4A, D, E**) displayed downregulated chromatin accessibility and mRNA levels in the Case group and restored in the Dapa group. These genes were further analyzed due to their significant correlation with the clinical features of DKD (**Figure 5**).

We first noticed *Isg15*, a pro-inflammatory gene, as functional enrichment analysis of RNA sequencing highlighted interferon-related pathways (**Figure 2**). In the current study, the univariate analysis including all mice from three groups demonstrated that *Isg15* expression levels were positively correlated to hyperglycemia and UACR (**Figures 5A, B**), suggesting its deleterious role in DKD. We also found positive correlations between another pro-inflammatory gene, *Csf2rb* mRNA levels in the kidney and the blood glucose concentration, serum urea nitrogen levels, and UACR of diabetic mice (**Figures 5C–E**), implying that CSF2RB should be detrimental in DKD. On the contrary, the anti-inflammatory gene *Btla* presented a negative correlation to serum creatinine concentration and UACR (**Figures 5F–H**). Therefore, we concluded that DKD is associated with renal inflammation, as pro-inflammatory cytokines would exacerbate while anti-inflammatory might attenuate kidney injury.

### Dapagliflozin regulated the expression of candidate genes and attenuated renal inflammation in type 2 diabetic mice *in vivo*

**Figure 6:**
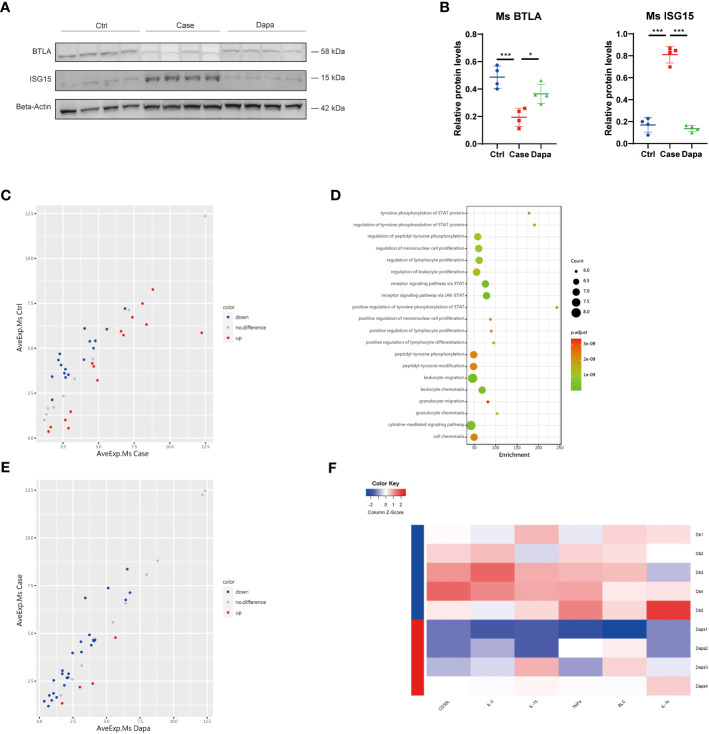
Dapagliflozin regulated the expression of candidate genes and attenuated renal inflammation in type 2 diabetic mice in vivo. **(A)** Western blot analysis showing BTLA and ISG15 protein levels in normal (Ctrl), diabetic (Case), and dapagliflozin-treated diabetic (Dapa) mouse kidneys, with corresponding quantifications in **(B)**. **(C)** Scatter plot indicating distinct protein expression levels of normal versus diabetic mouse kidneys. **(D)** GO enrichment analysis suggesting leukocyte activation and JAK-STAT pathway activation in diabetic mouse kidneys. **(E)** Scatter plot indicating distinct protein expression levels of diabetic versus dapagliflozin-treated diabetic mouse kidneys. **(F)** Heatmap showing different protein levels of inflammatory cytokines in diabetic (Db) and dapagliflozin-treated diabetic (Dapa) mouse kidneys. *P < 0.05, ***P < 0.001, one-way ANOVA test.

Next, we tested these inflammation-related molecules at the protein level in the mice kidneys. Diabetic mice presented a significantly higher level of ISG15 than the normal controls, which was alleviated by the dapagliflozin treatment (**Figures 6A, B**). In contrast, reduced expression of the immunosuppressor BTLA was detected in the Case group that was restored by dapagliflozin administration (**Figures 6A, B**). Although CSF2RB was nearly undetectable at the protein level in the whole kidney lysates, these inflammatory proteins were consistent with their mRNA levels and the univariate analysis.

To further explore the effect of dapagliflozin on renal inflammation *in vivo*, we performed an inflammation-associated antibody array assay analysis of mice whole kidney lysates, and found significant differences in the protein components of mice kidneys from different groups. In comparison with the Ctrl group, diabetic mice presented both upregulated and downregulated proteins (**Figure 6C**). Gene ontology analysis revealed that the Case group displayed an apparent profile of inflammatory cell proliferation and activation, as well as JAK-STAT pathway activation (**Figure 6D**). In contrast, mice treated with dapagliflozin showed overall downregulated inflammatory proteins in the kidney lysates (**Figure 6E**). Dapagliflozin administration triggered significant downregulation of two well-characterized inflammatory markers, tumor necrosis factor-alpha (TNF-α) and interleukin-1beta (IL-1β) (**Figure 6F**). Furthermore, dapagliflozin reduced the expression of multiple pro-inflammatory molecules in the diabetic kidney: CD30L, also known as CD153, can bind CD30 to promote senescence-associated tertiary lymphoid tissue expansion, facilitating local T and B cell interactions in the chronically inflamed kidney (12); IL-3 can act as the ligand of the aforementioned receptor CSF2RB (13); IL-15 is a pluripotent cytokine that supports both myeloid immune cells and lymphocytes, regulating innate and adaptive immune system (14); B lymphocyte chemoattractant, also called CXCL13, plays a role in diabetic pain and production of pro-inflammatory cytokines IL-1β, TNF-α, and IL-6 (15). In summary, dapagliflozin administration attenuated the overall inflammatory profile of the diabetic kidney in mice.

### Dapagliflozin presented an anti-inflammatory and anti-fibrotic effect on human renal tubular cells independent of glucose concentrations *in vitro*

**Figure 7:**
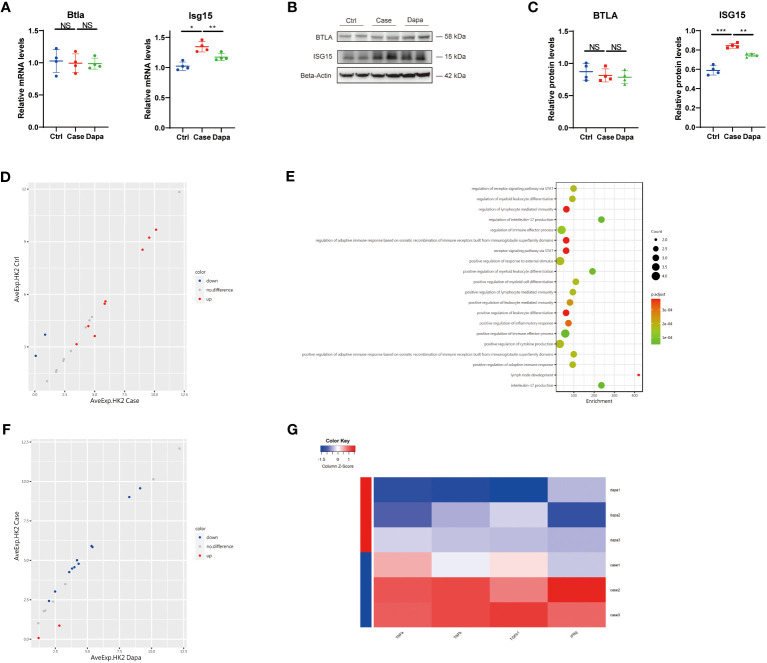
Dapagliflozin presented an anti-inflammatory and anti-fibrotic effect on human renal tubular cells independent of glucose concentrations in vitro. **(A)** QPCR analysis showing *Btla* and *Isg15* mRNA levels in HK-2 cells cultured in normal-glucose (Ctrl), high-glucose (Case), and high-glucose with dapagliflozin (Dapa) medium. **(B)** Western blot analysis showing BTLA and ISG15 protein levels in HK-2 cells of Ctrl, Case, and Dapa groups, with corresponding quantifications in **(C)**. **(D)** Scatter plot indicating distinct protein expression levels of HK-2 cells in Ctrl versus Case group. **(E)** GO enrichment analysis suggesting STAT pathway activation, inflammatory response, and leukocyte activation pathways in HK-2 cells in the high-glucose medium. **(F)** Scatter plot indicating distinct protein expression levels of HK-2 cells in Case versus Dapa group. **(G)** Heatmap showing different protein levels of inflammatory and fibrotic cytokines in HK-2 cells of Case and Dapa group. NS, not significant. *P < 0.05, **P < 0.01, ***P < 0.001, one-way ANOVA test.

With the aim of dissection of the renoprotective role of dapagliflozin at the cell level, we performed a cell culture of the human proximal tubular cell line (HK-2) with high expression of the target receptor SGLT2 (16). We found augmented mRNA levels of *ISG15* in tubular cells cultured in the high-glucose medium (Case group) versus cells cultured in normal-glucose medium (Ctrl group), which was alleviated by dapagliflozin treatment under the same high glucose concentration conditions (Dapa group) (**Figure 7A**). However, there was no expression of *CSF2RB* in proximal tubular cells, and the level of *BTLA* was kept almost unchanged regardless of the glucose or dapagliflozin treatment, indicating that dapagliflozin did not directly impact BTLA expression in renal tubular cells (**Figure 7A**). Correspondingly, immunoblot presented elevated ISG15 expression in the Case group that was attenuated in the Dapa group (**Figures 7B, C**). Subsequently, we performed an inflammation-associated antibody array assay analysis of the cell culture medium to further explore dapagliflozin’s effect on tubular cells under hyperglycemia. Renal tubular cells tended to produce more inflammatory molecules in the high-glucose medium (**Figure 7D**), activating the STAT pathway and promoting inflammation (**Figure 7E**). Incubation with dapagliflozin attenuated HK-2 secretion of the inflammatory proteins (**Figure 7F**), especially the well-known marker TNF-α and the fibrosis marker transforming growth factor-beta (TGF-β) into the cell culture medium (**Figure 7G**), implying an anti-inflammatory, anti-fibrotic role of dapagliflozin in the diabetic kidney. Moreover, we detected amelioration of two other well-characterized inflammation-associated molecules by dapagliflozin treatment, i.e., TNF-β and IFN-γ (**Figure 7G**). Of note, the aforementioned effects of dapagliflozin on HK-2 cells were independent of glucose concentrations, as the Case group and the Dapa group were cultured under the same glucose concentration conditions. We concluded that dapagliflozin served as an anti-inflammatory, anti-fibrotic agent to protect renal proximal tubular cells against DKD independent of glucose concentrations.

### A novel anti-inflammatory role of dapagliflozin in macrophages independent of glucose concentrations *in vitro*

**Figure 8:**
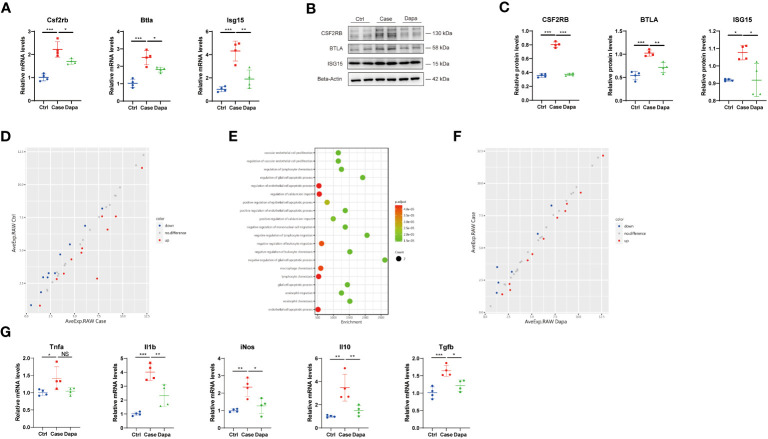
A novel anti-inflammatory role of dapagliflozin in macrophages independent of glucose concentrations in vitro. **(A)** QPCR analysis showing *Csf2rb*, *Btla*, and *Isg15* mRNA levels in RAW 264.7 cells cultured in normal-glucose (Ctrl), high-glucose (Case), and high-glucose with dapagliflozin (Dapa) medium. **(B)** Western blot analysis showing CSF2RB, BTLA, and ISG15 protein levels in macrophages of Ctrl, Case, and Dapa groups, with corresponding quantifications in **(C)**. **(D)** Scatter plot indicating distinct protein expression levels of macrophages in Ctrl versus Case group. **(E)** GO enrichment analysis suggesting endothelial cell apoptosis and leukocyte chemotaxis pathways in macrophages in the high-glucose medium. **(F)** Scatter plot indicating distinct protein expression levels of macrophages in Case versus Dapa group. **(G)** QPCR analysis showing *Tnf-α*, *Il-1β*, *iNos*, *Il-10*, and *Tgf-β* mRNA levels in macrophages of Ctrl, Case, and Dapa group. NS, not significant. *P < 0.05, **P < 0.01, ***P < 0.001, one-way ANOVA test.

As macrophages are considered the major leukocyte in the chronically inflamed kidney in diabetes (5), and our data highlighted the role of antigen-presenting cells and inflammation in DKD, we further investigated the role of dapagliflozin in macrophages. The immortalized mouse macrophage cell line (RAW 264.7) was cultured *in vitro*, and we detected upregulation of ISG15, BTLA, and CSF2RB in the high-glucose medium at both mRNA and protein levels (**Figures 8A, C**). Surprisingly, with our immunoblot analysis showing SGLT2 expression on macrophages (**Supplemental Figure 2D**), dapagliflozin treatment significantly alleviated the upregulation of these inflammatory molecules (**Figures 8A–C**). As macrophages might secret various cytokines to interact with multiple cell types, we also detected a series of molecules in the cell culture medium supernatant utilizing an inflammation-associated antibody array assay. Results of DEPs were enriched in the endothelial cell apoptosis process and leukocyte chemotaxis, suggesting that macrophages in hyperglycemia might induce endothelial injury and recruit other leukocytes to exacerbate inflammatory responses (**Figures 8D, E**). However, dapagliflozin treatment seemed to have little impact on the cytokines in the macrophage cell culture medium supernatant according to the antibody array assay. To deepen our investigations, we tested the role of glucose levels and dapagliflozin in macrophage polarization. High-glucose incubation activated macrophages to express a higher level of *Tnf-α*, *Il-1β*, *iNos*, *Il-10*, and *Tgf-β*, implying complicated macrophage activation and polarization process with a combination of different phenotypes (**Figure 8F**) (17). Dapagliflozin treatment attenuated the activation of these macrophages, exerting a seemly “calm down” effect (**Figure 8F**). Altogether, these results revealed a novel, unrecognized role of dapagliflozin in regulating inflammatory response via its effect on macrophages and independent of glucose concentrations, and its specific signaling pathways deserve further exploration.

### Dapagliflozin attenuated kidney injury and alleviated renal inflammation in an ischemia/reperfusion-induced mouse CKD model

**Figure 9:**
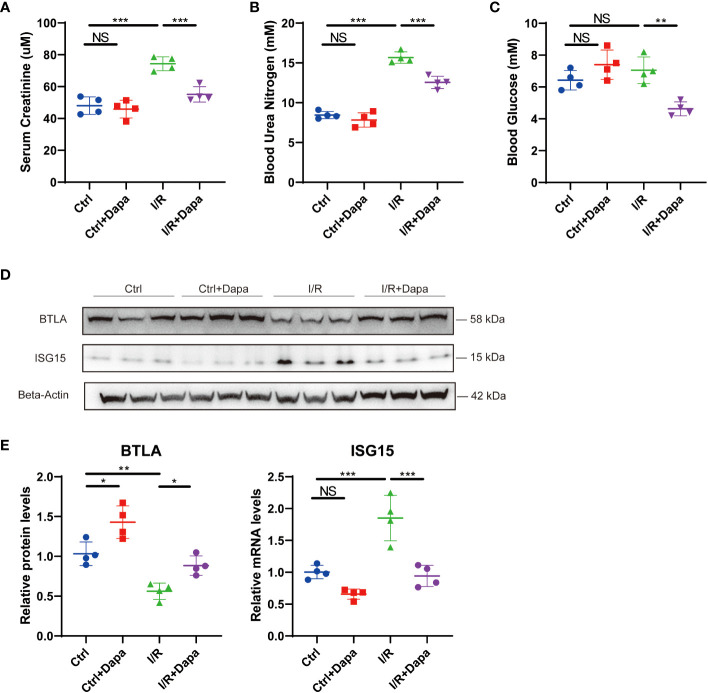
Dapagliflozin attenuated kidney injury and alleviated renal inflammation in an ischemia/reperfusion-induced mouse CKD model. **(A)** Serum creatinine levels of normal (Ctrl), dapagliflozin-treated normal (Ctrl+Dapa), ischemia/reperfusion-operated (I/R), and dapagliflozin-treated I/R (I/R+Dapa) 14-week-old mice. **(B)** Blood urea nitrogen levels of 14-week-old mice in the Ctrl, Ctrl+Dapa, I/R, and I/R+Dapa groups. **(C)** blood glucose levels of 14-week-old mice in the Ctrl, Ctrl+Dapa, I/R, and I/R+Dapa groups. **(D)** Western blot analysis showing BTLA and ISG15 protein levels in mouse kidneys in the Ctrl, Ctrl+Dapa, I/R, and I/R+Dapa groups, with corresponding quantifications in **(E)**. NS, not significant. *P < 0.05, **P < 0.01, ***P < 0.001, one-way ANOVA test.

Considering that our cell culture experiments suggested a blood glucose-independent, anti-inflammatory effect of dapagliflozin, we subsequently explored the potential role of dapagliflozin in healthy mice and mice with CKD induced by ischemia/reperfusion (I/R). Dapagliflozin administration in healthy mice caused no apparent changes in serum creatinine (48.05 ± 4.7 µM in Ctrl versus 45.88 ± 4.9 µM in Ctrl+Dapa, P=0.9294), blood urea nitrogen (8.45 ± 0.4 mM in Ctrl versus 7.83 ± 0.8 mM in Ctrl+Dapa, P=0.6332), and blood glucose levels (6.43 ± 0.5 mM in Ctrl versus 7.40 ± 0.8 mM in Ctrl+Dapa, P=0.2799) (**Figures 9A–C**). At 7 days post I/R surgery, serum creatinine (74.35 ± 3.8 µM in I/R, P<0.0001) and blood urea nitrogen levels (15.68 ± 0.6 mM in I/R, P<0.0001) elevated while blood glucose (7.05 ± 0.7 mM in I/R, P=0.6293) remained unaltered in CKD mice compared with Ctrl mice. In contrast, treatment with dapagliflozin significantly restored kidney function (serum creatinine=55.15 ± 4.2 µM in I/R+Dapa, P=0.0009 compared with I/R; blood urea nitrogen=12.55 ± 0.7 mM in I/R+Dapa, P=0.0003 compared with I/R) and reduced blood glucose levels (4.63 ± 0.4 mM in I/R+Dapa, P=0.0024 compared with I/R) in I/R-induced CKD mice (**Figures 9A–C**). Then, we detected the protein levels of BTLA and ISG15 in the mice kidneys. Compared with the Ctrl group, healthy mice treated with dapagliflozin displayed augmented BTLA expression, and their ISG15 expression seemed to decrease but did not reach a significant threshold, probably due to the low ISG15 protein levels at the baseline (**Figures 9D, E**). Furthermore, I/R surgery downregulated BTLA while upregulated ISG15 in the kidney, and such a pro-inflammatory state was ameliorated by dapagliflozin (**Figures 9D, E**). As a result, dapagliflozin played a renoprotective, anti-inflammatory role in I/R-induced CKD mice.

## Discussion

Utilizing integrated bioinformatic, *in vivo*, and *in vitro* approaches, we have revealed the inflammatory profile of the chronically diseased kidney in diabetes and how SGLT2 inhibitor dapagliflozin could ameliorate DKD. First, we noticed that the immune system showed significant alterations in DKD and was impacted by dapagliflozin administration. Next, with the help of RNA sequencing and ATAC analysis, we identified three key inflammation-related genes, i.e., *Csf2rb*, *Btla*, and *Isg15*, whose mRNA levels were correlated to the severity of DKD. When detecting the protein levels of these molecules, we found that in accordance with the RNA sequencing results, diabetic mice upregulated the pro-inflammatory molecules while downregulated the anti-inflammatory protein BTLA. Furthermore, we dissected the impact of hyperglycemia and the effect of dapagliflozin at the cell level. We demonstrated that high glucose concentration induced inflammatory molecules in renal proximal tubular cells and macrophages, which could be alleviated by dapagliflozin treatment independent of glucose concentrations. Our findings would contribute to the mechanical understanding of DKD and provide novel evidence for the therapeutic effect of dapagliflozin.

In the current study, we based on the bioinformatic analysis of RNA sequencing and ATAC analysis data to screen for key genes that were correlated to the clinical features of DKD and could interact with the anti-hyperglycemic agent dapagliflozin. Intriguingly, the three candidate genes we found were all associated with inflammation: CSF2RB, also known as cytokine receptor common subunit beta, encodes the common β subunit to the receptor of common chain cytokines granulocyte-macrophage colony-stimulating factor (GM-CSF), IL-3, and IL-5 (13). In addition to the function of hematopoiesis, these cytokines play a pivotal role in inflammatory responses (13, 18), as is the case of chronic microvascular inflammation in DKD. ISG15 is a ubiquitin-like protein that can either be secreted as a free protein or bound target protein to induce a reversible post-translational modification called ISGylation (19). ISG15 exerts multiple effects, involving anti-infectious responses (19), metabolic reprogramming (20), and inflammatory responses (21). BTLA is an inhibitory receptor that suppresses immune responses, and its expression might be associated with poor prognosis in tumors (22), fulminant viral hepatitis (23), and sepsis (24). The pro-inflammatory molecules CSF2RB and ISG15 were positively correlated with kidney injury parameters, while the anti-inflammatory molecule BTLA displayed a negative correlation. Hence, our study shed light on the role of inflammatory factors in DKD. Another finding in our study was elevated antigen processing and presentation in diabetic mice, with both MHC class I and class II upregulated. It has been reported that type 1 diabetes mellitus as an autoimmune disease is associated with certain MHCs (25). Nevertheless, in metabolic type 2 diabetes mellitus, as in our mouse model, the role of antigen processing and presentation remains largely unclear. The kidney is an organ with an abundant blood supply and thus various leukocytes, and augmented antigen processing and presentation in DKD could be associated with chronic renal inflammation and injury (26). Further investigations should explore the specific autoantigen in DKD since it is a non-infectious disease, the major activated antigen-presenting cells, and their downstream pathways.

We further explored the therapeutic effect of dapagliflozin on DKD. It has been reported that dapagliflozin as an SGLT2 inhibitor can reduce hyperglycemia and provide cardiovascular and renal benefits (27). Indeed, dapagliflozin administration in our research improved kidney function and albuminuria in diabetic mice, presenting a potent renoprotective effect. As for the mechanism by which dapagliflozin could protect against DKD, previous studies illustrated its effect on inhibition of HIF-1α-mediated renal tubular cell metabolic disorders (28), amelioration of renal tubular ferroptosis (29), and alleviation of complement over-activation (30). Here, we demonstrated that dapagliflozin attenuated renal inflammation in diabetic mice, downregulating a variety of inflammatory cytokines, including TNF-α, IL-1β, CD30L, IL-3, IL-15, and CXCL13. It has been recognized that chronic microvascular inflammation represents one key pathophysiological feature of diabetes mellitus (31); our results unraveled several key inflammatory molecules that participate in DKD, and demonstrated that dapagliflozin could attenuate renal inflammation by regulating the expression of multiple inflammatory molecules. Furthermore, in the current study, we focused on the effect of dapagliflozin on alleviating renal inflammation, and previous studies have demonstrated that dapagliflozin downregulated proteins involved in various organs and pathways: regarding metabolic changes, feeding with dapagliflozin suppressed glucagon signaling and reduced hepatic glucose production in rodent diabetic models (32); as for oxidative stress and cell death, dapagliflozin treatment downregulated hypoxia inducible factor 1α and heme oxygenase 1, thereby ameliorating ferroptosis in mouse DKD (33); besides, dapagliflozin inhibited fibrotic markers type I collagen, fibronectin, and α-smooth muscle actin in mouse myocardial damage (34). Overall, the therapeutical benefits of dapagliflozin should be comprehensive, involving multiple organ systems and signaling pathways, and deserve further investigations.

More precisely, to determine which cluster of cells directly responds to dapagliflozin treatment, we performed cell culture of two major cell types, i.e., renal tubular cells as the local intrinsic cells and macrophages as the infiltrated inflammatory cells. In proximal tubular cells, in addition to ISG15, we detected the reduction of TNF-β and IFN-γ by dapagliflozin administration. TNF-β, also known as lymphotoxin-α, plays a crucial role in lymphoid organ development, whose receptor has been found on lymphocytes and tubular epithelial cells to participate in renal inflammation and injury (35, 36). IFN-γ, except for its antiviral function, could induce inflammation, oxidative stress, and fibrosis of the kidney (37, 38). Such findings demonstrated that dapagliflozin successfully ameliorated inflammation and fibrosis caused by high glucose concentration, which was consistent with other researchers (39). To our surprise, dapagliflozin exerted a potent anti-inflammatory effect on cultured macrophages. It has been reported that dapagliflozin can mediate M2 polarization and modulate NLRP3 inflammasome activity of macrophage *in vivo* to induce cardioprotective and hepatoprotective effects (40–42). The novelty of our study resides in demonstrating that dapagliflozin can directly effect on macrophages to suppress inflammatory markers independent of glucose concentrations.

Based on our findings that dapagliflozin can attenuate renal inflammation independent of glucose concentrations, we then explored its role in Ctrl mice and I/R-induced CKD mice. We found that dapagliflozin treatment elevated BTLA expression and seemed to reduce ISG15 expression in Ctrl mice. In I/R-induced CKD models, decreased BTLA protein levels and increased ISG15 expression were observed in the diseased kidney. Surprisingly, dapagliflozin rescued kidney function, restored BTLA expression, and alleviated ISG15 upregulation. Of interest, dapagliflozin treatment in normal mice did not lower the blood glucose, while it decreased the blood glucose levels in I/R-induced CKD mice, and the mechanism underlying such a discrepancy deserves exploration. Since mice in the Ctrl and I/R group had no hyperglycemia but involved inflammation, these data further supported our conclusions, and provided new targets for the intervention of CKD using dapagliflozin.

There are several limitations of our study. First, we successfully proved that dapagliflozin could attenuate inflammation in the whole kidney, renal tubular cells, and macrophages, but there are still a group of other cell types in the kidney, such as mesangial cells, podocytes, and interstitial cells. Further investigations could take advantage of single-cell multi-omic technologies to acquire a more precise inflammatory profile of the diseased kidney in DKD with high resolution. In addition, although we proved that both HK-2 cells and RAW 264.7 cells expressed SGLT2, we failed to knock down SGLT2 protein levels utilizing siRNA (**Supplemental Figure 2**). SGLT2 knockout or knockdown mice or cell lines should be explored in the future to determine whether our observed changes are caused by SGLT2 inhibition or the off-target effect of dapagliflozin. Moreover, we separately explored the direct effect of dapagliflozin on HK-2 cells and RAW 264.7 cells. However, the interactions between renal cells and immune cells play pivotal roles from acute kidney injury to CKD, covering renal inflammation, regeneration, and fibrosis (43–45). Therefore, a co-culture system of both renal cells and immune cells from the same species could help to illustrate such delicate intercellular crosstalk and the possible effect of dapagliflozin.

In conclusion, our data demonstrate that dapagliflozin attenuates renal inflammation to protect against DKD, and prove that dapagliflozin can directly affect, in a way independent of blood glucose regulation, renal proximal tubular cells and macrophages as an anti-inflammatory agent. Our analysis emphasizes the role of inflammatory factors in the pathogenesis of DKD, and reveals novel therapeutic targets of dapagliflozin treatment. In that sense, this study contributes to the mechanistic understanding of DKD pathophysiology, and provides new insights into the renoprotective effects of dapagliflozin.

## Data availability statement

The data presented in the study are deposited in the GEO repository, accession number GSE246576 for RNAseq data and GSE246763 for ATACseq data.

## Ethics statement

Ethical approval was not required for the studies on humans in accordance with the local legislation and institutional requirements because only commercially available established cell lines were used. The animal study was approved by The ethics committee of Renji Hospital affiliated to Shanghai Jiao Tong University School of Medicine. The study was conducted in accordance with the local legislation and institutional requirements.

## Author contributions

XC, SM, and LG prepared the concept and designed the study. AC, JS, XY, XS, and XC performed the experiments. AC, JS, and XY analyzed data. AC and JS prepared the figures. AC, JS, and XY drafted the paper. XC, SM, and LG edited and revised the paper. AC, JS, and XY contributed equally to this paper. All authors contributed to the article and approved the submitted version.
