# Supplementary material for: Dapagliflozin alleviates renal inflammation and protects against diabetic kidney diseases, both dependent and independent of blood glucose levels
Source: Front Immunol. 2023 Nov 9;14:1205834. doi: 10.3389/fimmu.2023.1205834 (PMC10665888; doi:10.3389/fimmu.2023.1205834)
Supplement: Supplementary file 1 [file DataSheet_1.docx]

## Dapagliflozin alleviates renal inflammation and protects against diabetic kidney diseases, both dependent and independent of blood glucose levels

Anxiang Cai^1#^, Jianxiao Shen^1#^, Xiaoqian Yang^1#^, Xinghua Shao^1^, Leyi Gu^1*^, Shan Mou^1*^ and Xiajing Che^1*^

^1^ Department of Nephrology, Molecular Cell Lab for Kidney Disease, Shanghai Peritoneal Dialysis Research Center, Ren Ji Hospital, Uremia Diagnosis and Treatment Center, Shanghai Jiao Tong University School of Medicine, Shanghai 200127, China.

**^#^** **These authors contributed equally to this work and share first authorship**

**^#^Correspondence:**

Dr. Xiajing Che, Email: chexj@126.com;

Dr. Shan Mou, Email: shan_mou@shsmu.edu.cn

Dr. Leyi Gu, Email: guleyi@aliyun.com

**Keywords: diabetic kidney disease, sodium-glucose cotransporter 2 inhibitors, inflammation, renal tubular cell, macrophage.**


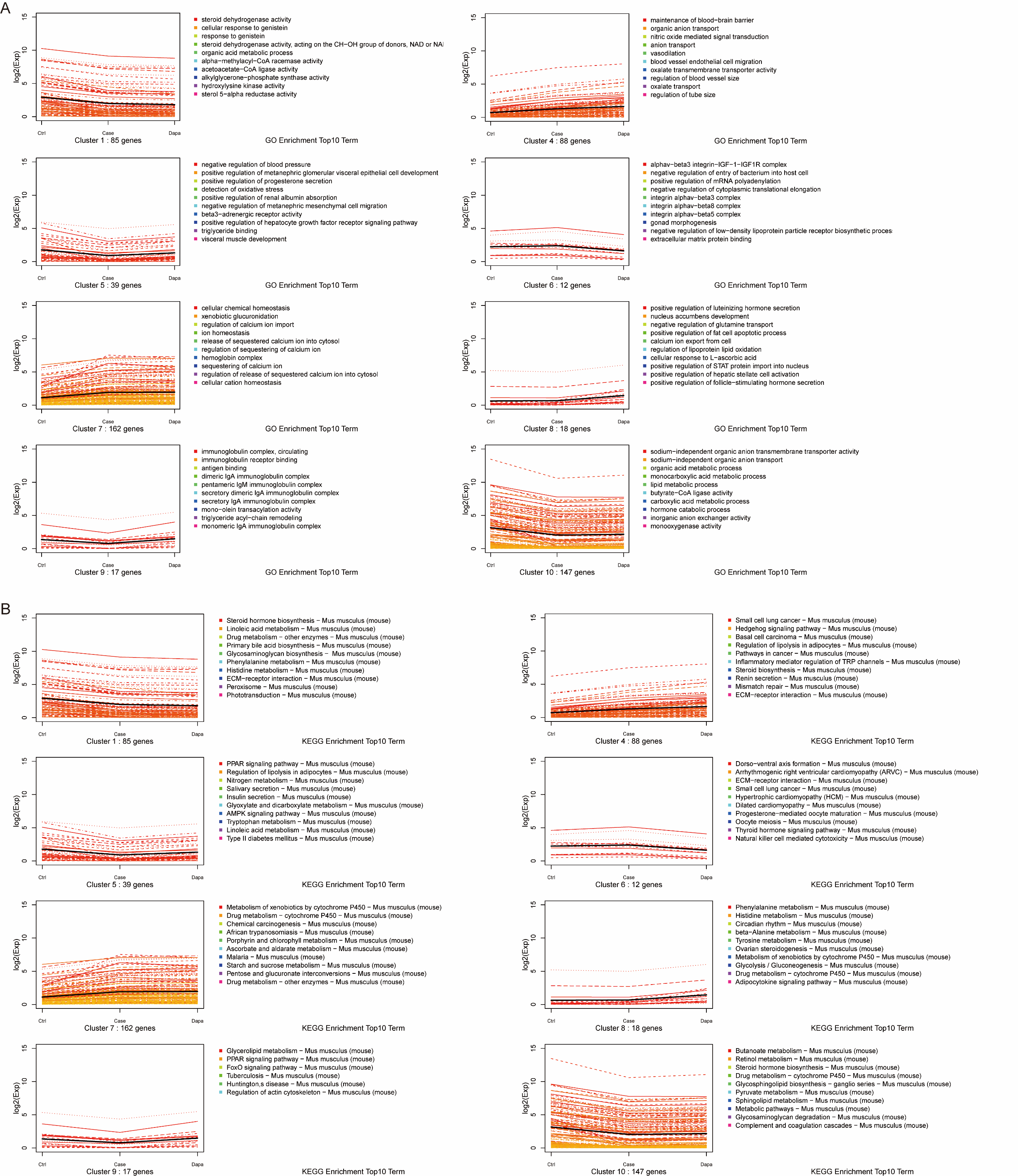
*Supplemental Figure 1. K-means analysis of RNA sequencing results*

K-means analysis yielded 10 clusters with different expression patterns among Ctrl, Case, and Dapa groups. (A) Expression patterns of genes in Cluster 1 and 4-10 with the top 10 enriched pathways listed according to GO terms. (B) Expression patterns of genes in Cluster 1 and 4-10 with the top 10 enriched pathways listed according to GO terms.


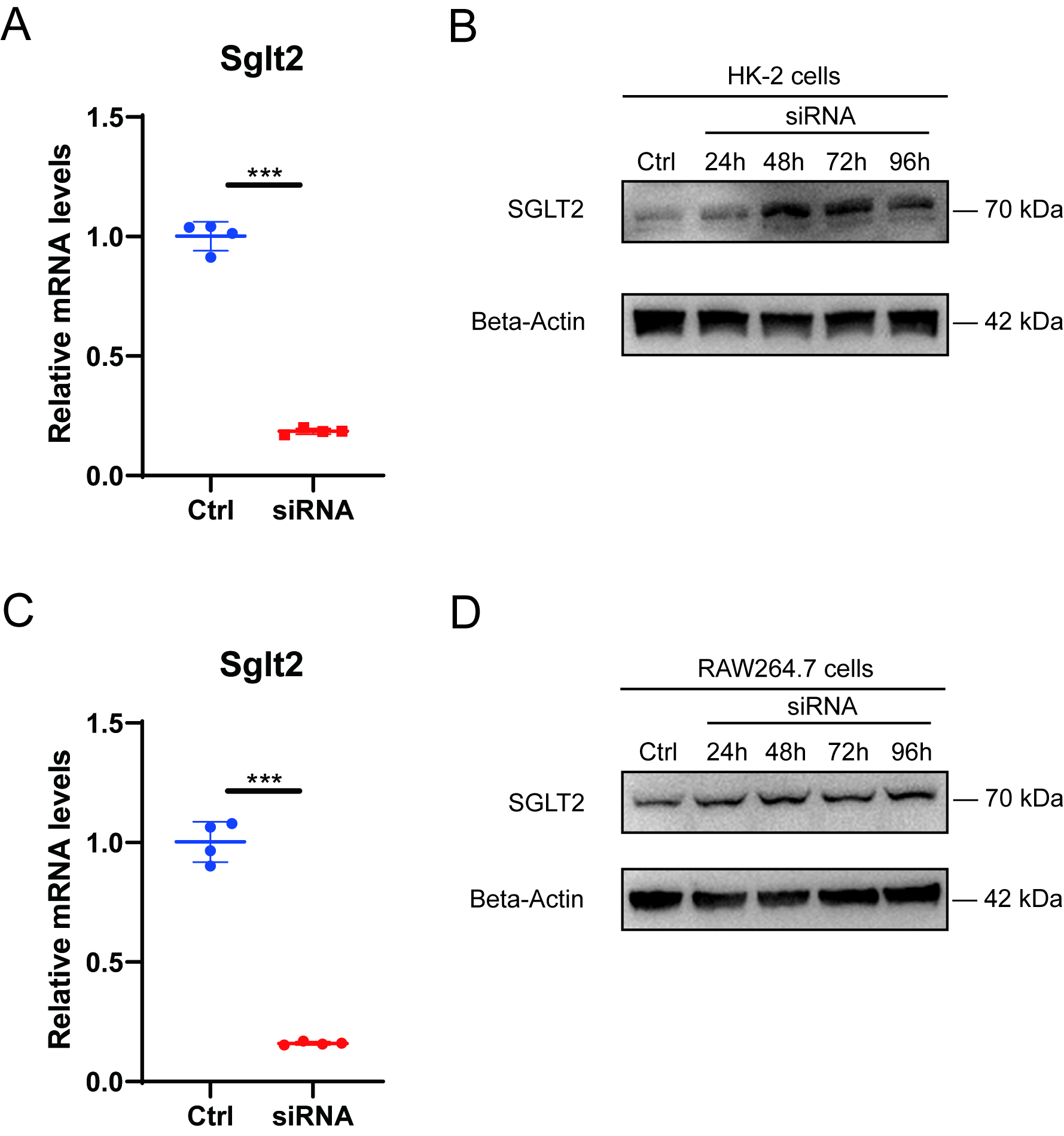


*Supplemental Figure 2. Expression and knockdown of SGLT2 in HK-2 and RAW 264.7 cells in vitro*

(A) QPCR analysis showing *Sglt2* mRNA levels in HK-2 cells from normal (Ctrl) group or siRNA-treated for 48 hours. (B) Western blot analysis showing SGLT2 protein levels in HK-2 cells of Ctrl group, and siRNA-treated for different durations. (C) QPCR analysis showing *Sglt2* mRNA levels in RAW 264.7 cells from normal (Ctrl) group or siRNA-treated for 48 hours. (D) Western blot analysis showing SGLT2 protein levels in RAW 264.7 cells of Ctrl group, and siRNA-treated for different durations.
